# Evaluation of an Application for Mobile Telephones (e-12HR) to Increase Adherence to the Mediterranean Diet in University Students: A Controlled, Randomized and Multicentric Study

**DOI:** 10.3390/nu14194196

**Published:** 2022-10-08

**Authors:** Luis M. Béjar, María Dolores García-Perea, Pedro Mesa-Rodríguez

**Affiliations:** 1 Department of Preventive Medicine and Public Health, School of Medicine, University of Seville, 41004 Seville, Spain; 2 Virgen Macarena University Hospital, 41009 Seville, Spain; 3 Camas Health Center, 41900 Seville, Spain

**Keywords:** Mediterranean diet, dietary assessment, Mediterranean diet score, Mediterranean diet adherence, mobile applications, food, information and communication technologies

## Abstract

Mediterranean diet (MD) is potentially one of the best diets regarding health benefits and sustainability. However, it is faced with serious difficulties staying alive, even in traditionally Mediterranean regions. The objective was to evaluate the effectiveness of an application (e-12HR) to improve adherence to the MD (AMD) in university students. This study was a controlled, randomized, and multicentric clinical trial with two parallel groups (control group (CG) and intervention group (IG)), a 28-day follow-up period, and 286 participants (74.1% women). There were two versions of e-12HR: ‘feedback’ e-12HR (IG) and ‘non-feedback’ e-12HR (CG). Only the ‘feedback’ e-12HR had two specific automatic functions: 1. Evaluation of the user’s AMD; 2. Identification of the food groups for which the user has not fulfilled the MD recommendations. Both versions of the application allowed the collection of data on dietary intake in order to calculate the AMD. When comparing CG and IG at 14-, 21-, and 28-days follow-up (no significant statistical differences at baseline), there were significant statistical improvements in favor of IG in AMD index (0.71, 1.56, and 1.43 points, respectively), and in the percentage of participants with medium/high AMD index (14.4%, 20.6%, and 23.7%, respectively). In conclusion, e-12HR could improve AMD among university students.

## 1. Introduction

In 2019, around the world, there were 55.4 million deaths (41 million from non-communicable diseases) (NCD) [1]; and of these, 8.0 million were as a result of dietary risks [2]. As such, the prevention and treatment of NCDs related to diet is a topic of pressing global concern [3].

The World Health Organization’s (WHO) Global Action Plan for the Prevention and Control of NCDs 2013–2020 [4] recognizes the fundamental importance of reducing the degree of exposure for people and populations to common behavioral (and thus modifiable) risk factors for NCDs (although, this study was focused on dietary risk factors). The Mediterranean diet (MD) (characterized by the regular intake of olive oil and plant-based foods—cereals, fruits, vegetables, legumes, and nuts—the moderate consumption of fish and milk products, the low to moderate consumption of alcohol—mainly red wine—and the low intake of red or processed meat [5]) is a dietary pattern at once healthy and sustainable; healthy: more and more evidence supports the role of the MD, regarding primary and secondary prevention, protecting against cardiovascular disease, cancer, diabetes mellitus, arteriosclerosis, metabolic syndrome, respiratory disease (asthma and sleep apnea), excess weight/obesity, mental disorders (cognitive decline and depression), as well as renal diseases [6,7,8,9,10]; sustainable: the MD is also known for its low environmental impact, and its rich biodiversity, a high sociocultural value is placed on the foods, and the diet benefits local economies [10,11,12,13,14,15,16]. All of which makes it, without a doubt, an enticing alternative for reducing the degree of exposure to dietary risk factors for NCDs, although, to benefit from this, high adherence to the MD (AMD) is obviously necessary (AMD is a metric which provides an assessment of the degree to which individuals follow the MD, so that, except in a few exceptions, the highest scores indicate high ADM [17,18]). However, the MD (with its health benefits and sustainability) faces great difficulties regarding its implementation in other geographic and cultural regions, and it is even struggling to stay relevant, surprisingly, in traditionally Mediterranean regions (which is influenced by unhealthy habits resulting from global acculturation) [8] where it is being abandoned, mainly by younger generations [15]. 

The WHO’s Global Action Plan for the Prevention and Control of NCDs 2013–2020 [4] recognizes, as well, the fundamental importance of strengthening the abilities of people and populations to make healthier decisions and to adopt behaviors that foster better health (in this study, in relation to the adoption of the MD). Indeed, as part of the dietary assessment process, healthcare professionals seek to evaluate individual dietary patterns in order to suggest corrective actions and transform them into healthier ones [3].

Ultimately, to prevent and treat many nutrition-related NCDs, it is necessary to maintain adequate dietary patterns [3,4]. For this, it is crucial to have: 1. A precise assessment of dietary intake [3] (in the present study, this is essential for calculating AMD; not knowing one’s AMD makes it difficult for individuals to follow the MD and complicates the dissemination of such a healthy and sustainable dietary pattern [19]); 2. A proposal for corrective actions to improve diet [3] (in this case, to reach a higher ADM index). For this reason, the research team has developed an application for mobile telephones (e-12HR) with two specific automatic functions: (1) Evaluation of the user’s AMD index; 2. Identification of the food groups for which the user has not fulfilled the MD recommendations (hereafter referred to as ‘non-compliant’ food groups). In addition, e-12HR also provided the user with the consumption recommendations established for these ‘non-compliant’ food groups (see e-12HR App, (2) Feedback From e-12HR subsection: AMD Report). This information could be considered as corrective actions for improving AMD. 

e-12HR is a previously validated application for determining the habitual consumption of food groups [20,21,22,23,24]; however, by adding some simple algorithms, it has been possible to expand its development with the two new functionalities mentioned above.

The main hypothesis of this study was that the use of e-12HR with the two new functionalities (hereafter referred to as ‘feedback’ e-12HR) among Spanish university students (a group with low AMD index and one of the groups of the younger generations that is abandoning the MD [25]), could have an influence on improving AMD index. To the best knowledge of the research team, this has been the first study that has evaluated an application for mobile telephones to increase AMD among university students. The primary objective of this research was to evaluate the effectiveness of using ‘feedback’ e-12HR, intervention group (IG), for 28 days, versus the use of e-12HR without the two new functionalities (hereafter referred to as ‘non-feedback’ e-12HR), control group (CG), in increasing AMD index. A secondary objective was to assess the application’s usability.

## 2. Materials and Methods

### 2.1. Study Design

This study is a controlled, randomized, and multicentric clinical trial with two parallel groups and a follow-up period of 28 days.

### 2.2. Participants

Participants (university students) were included by probability single-stage cluster sampling at the Faculties of Medicine and Pharmacy at the University of Seville (Andalusia, Spain, South of Europe). Four random classrooms were selected in each school.

In each of the selected classrooms, the students were informed of the objectives, risks, and benefits of the research, including collection of samples. Confidentiality of participant data was guaranteed at all times in accordance with the provisions of the Organic Law on the Protection of Personal Data (15/1999 of 13 December, LOPD) and under the conditions established by Law 14/2007 of Spanish biomedical research.

Inclusion criteria: Both genders, over the age of 18, students of Medicine or Pharmacology at the Faculties of Pharmacy or Medicine (University of Seville, Seville, Spain), and own a mobile phone with Internet access and an iOS or an Android operating system. 

Exclusion criteria: Food intolerances, chronic pathologies, or pregnancy (due to the possibility of requiring specialized dietary recommendations).

A member of the research group showed the project to the participants and provided information on how to participate in the study (interested students must send an e-mail to the study address provided in the presentation) and how the e-12HR app works. Once an e-mail was received from the interested students, a member of the research team responded with the following documents and information: (1) Informed consent to be signed by the student and returned to the same e-mail address; (2) A form with personal information such as gender, date of birth, where they study, weight, height, smoking status) to be completed by the student and returned to the same e-mail address; (3) A personally assigned alphanumeric code; (4) Instructions for downloading the e-12HR app, which is free to download from the App Store (for iOS) or Play Store (for Android); (5) A user manual with detailed information for using the e-12HR app; (6) A brochure with the recommendations for consumption by food groups of the MD. 

The procedure was enacted to increase study participation, avoid unnecessary travel in order to sign or fill in documents, and promote conservation efforts by not using paper.

Participant recruitment took place in April 2022.

Study participation was incentivized with the possibility of winning school materials (valued at EUR 500) in a raffle among the participants who successfully completed the study. 

### 2.3. Randomization and Masking

Of the 4 selected classrooms in each school (Medicine and Pharmacy), 2 were randomly assigned to the CG and 2 to the IG.

Due to the nature of the intervention, the participants could not be blinded. However, the person responsible for the statistical analysis remained blinded throughout the study. In addition, although the different versions of the application were available in the Apple Store or Play Store at all times, as previously mentioned, personal alphanumeric codes were assigned so that each participant only had access to their version of the application depending on whether they belonged to the IG (‘feedback’ e-12HR, with the two new functionalities) or to the CG (‘non-feedback’ e-12HR, without the two new functionalities).

**Figure 1:**
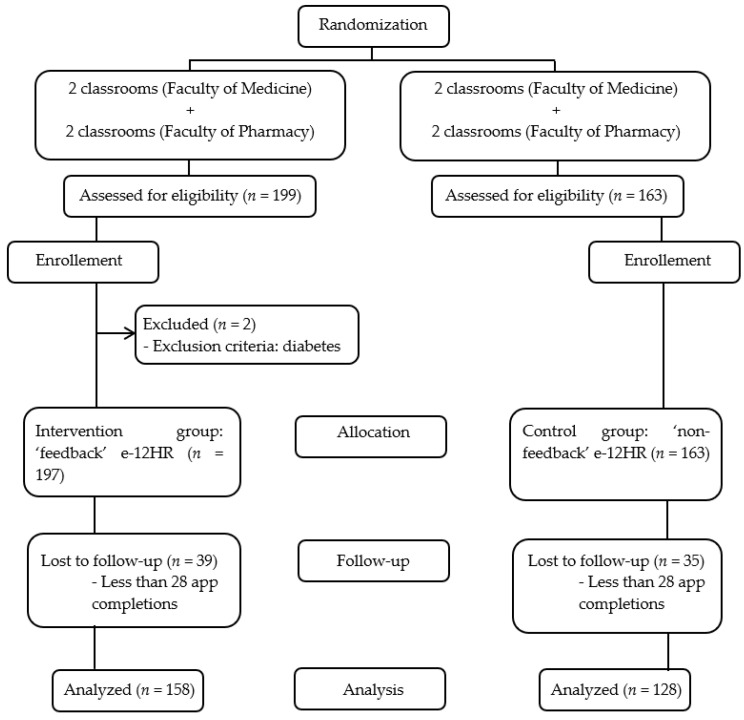
Flowchart of the Study.

The allocation sequence is shown in Figure 1.

### 2.4. Intervention

IG: the participants used ‘feedback’ e-12HR for 28 days.

CG: the participants used ‘non-feedback’ e-12HR for the same time period.

e-12HR App

1. Data Collection on Food Group Intake

The operation of e-12HR is similar for the two versions of the application used in the present study (‘feedback’ e-12HR and ‘non-feedback’ e-12HR).

e-12HR is an application that has been previously validated and that allows for collecting long-term data on dietary intake of food groups [20,21,22,23,24].

Once downloaded, participants were asked to activate the app on first use by typing in a personally assigned alphanumeric code. After this, participants would register the number of standard portions of each of the 19 food groups from the study that they consumed during the day. These food groups were vegetables, breakfast cereals, fruits, pasta, rice, bread, olive oil, milk and dairy products, nuts, fermented beverages (beer and wine), potatoes, fish, white meat, legumes, eggs, red meat, processed meats, and sweets. The app lets users register in minutes how much moderate and intense physical activity they had performed throughout the day.

Participants were also instructed to use the app once they had finished their last meal of the day [26,27]. The app can be completed only between 8 PM and 4 AM, which might seem to be a strange time range, but the team chose it in order to give users enough time to complete the task. It was taken into account that university students, who are mainly young adults, tend to go out on evenings and can stay out eating and drinking until late at night. The time period selected allowed users to register those foods/drinks in the application, as well. 

At the end of each monitoring day, an alert appeared on the participants’ phones, letting them know that it was time to complete the app. Participants were allowed to set the time for the alert according to their own preferences). From that point, participants could access the task and enter the number of standard servings they had consumed during that day for each of the food groups mentioned above and the amount of physical activity in minutes. 

**Figure 2:**
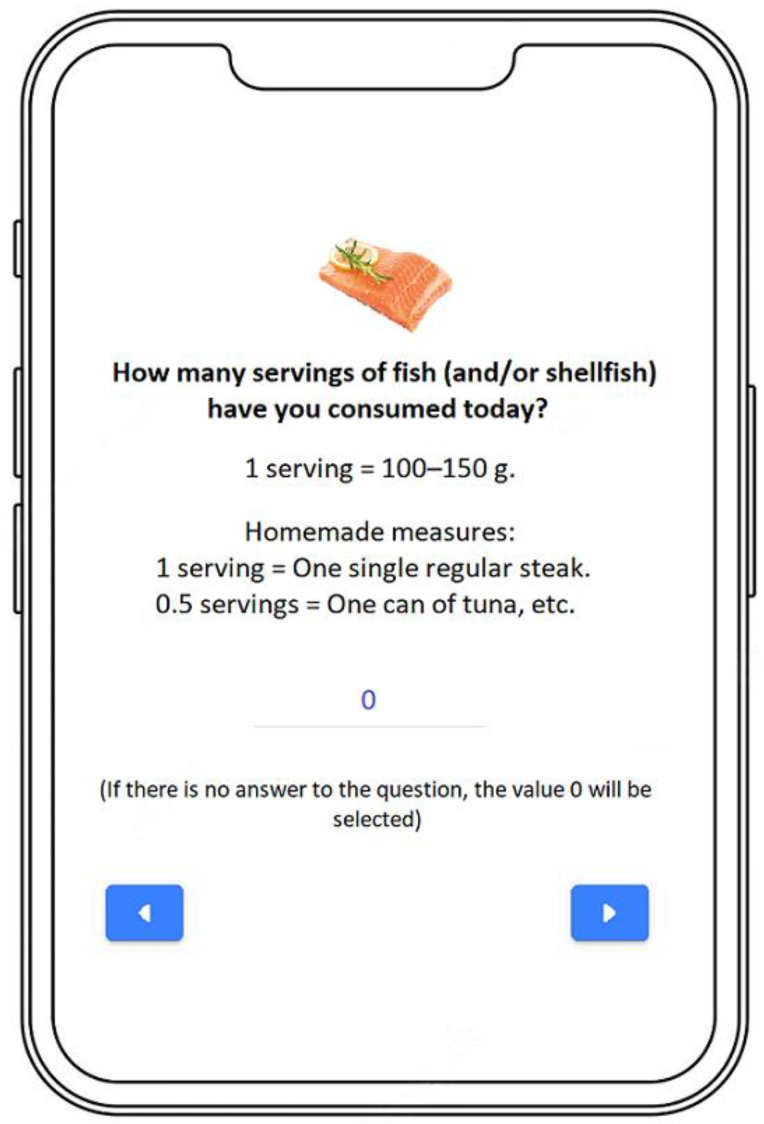
Screenshot from the e-12HR App.

In order to assist with the estimation of the number of servings consumed, each food group was accompanied by a text that explained different homemade measures since the research team considered it more appropriate and easier to follow for participants without prior experience in dietetics. The standard servings used by e-12HR were based on a previously validated semiquantitative food frequency questionnaire for people in Spain [28]. As an example, participants would see the following when using the app: “How many servings of fish (and/or shellfish) have you consumed today? 1 serving = 100–150 g. Homemade measures: 1 serving = One single regular steak. 0.5 servings = One can of tuna, etc.”. Participants introduced the corresponding number in the “Answer” section and then tapped the “Next” button to go on to the next food group. The app also lets participants use decimals to estimate the portions consumed. If there was an error when registering information, participants were able to return to the previous page by tapping the “Previous” button to begin the process again (Figure 2).

To improve the app’s usability, the different food groups were in the same order every day (Appendix A, Table A1) and each food group had a representative image.

After completing the daily questionnaire, the information collected was automatically saved in the participant’s mobile phone (to be used later by the application, see 2. Feedback From e-12HR subsection) and sent to the website of the study administrators. This meant that the participants were not able to change their previous responses, and they were not able to access the app until the following day when they were prompted to complete the next questionnaire. 

Dietary data registration through the e-12HR app was scheduled for 28 consecutive days. Study participants could see when they had completed the study period as there was a counter with the number of days the task had been successfully completed in the app.

2. Feedback from e-12HR

This section is only applicable to ‘feedback’ e-12HR app.

AMD Assessment

Every seven days during the 28-day study period, as an automatic function, the application processed the daily data collected on the user’s food intake in order to establish an AMD index, specifically, the Mediterranean Diet Serving Score (MDSS) index [29]. To do so, previously-established rules were taken into account, which considers [29]:

1. The specific food groups (compatible with the MD);
2. Consumption recommendations for frequency of standard servings (per meal, daily or weekly);
3. A numerical score assigned to each item (Appendix A, Table A2).

However, in the case of recommendations on frequency of consumption, some modifications had to be made in order to adapt them to the characteristics of e-12HR [25] (Table 1).

Cereals: pasta, breakfast cereals, bread and rice. Olive oil: for salads, on bread or for frying. Milk and dairy products: yogurt, cheese and milk. Fermented beverages such as wine and beer. White meat: poultry. Red meat: beef, pork and lamb. Sweets: candies, sugar, pastries, sweetened fruit juices and soft drinks.

For food groups calculated on a weekly basis, the app used the rules shown in Table 1. However, for food groups calculated on a daily basis, the procedure was as follows: every seven days, for each specific food group, the number of standard portions registered daily throughout this period were added all together first, and the result was then divided by seven. For the food groups calculated on a daily basis with scores greater than 1, the scoring rules were as follows [19,25]:

1. Fruits contribute 1 point for 1—<2 servings, 2 points for 2—<3 servings, and 3 points for 3—6 servings per day. 

2. Vegetables contribute 1 point for 2—<4 servings, 2 points for 4—<6 servings, and 3 points for ≥6 servings per day. 

3. Cereals contribute 1 point for 1—<2 servings, 2 points for 2—<3 servings, and 3 points for 3—6 servings per day.

4. Olive oil contributes 1 point for 1—<2 serving, 2 points for 2—<3 servings, and 3 points for 3—4 servings per day.

5. Milk and dairy products contribute 1 point for 1—<2 servings and 2 points for 2—3 servings per day.

6. Nuts contribute 2 points for 1—2 servings per day. 

For each individual food group, if the indicated recommendations are not followed, the app assigns a value of zero for that group. 

To complete the process, e-12HR added up all of the values and released a scoring of the AMD index (MDSS index), which could vary between zero and twenty-four.

On top of this, as an automatic function, the app related the score on the AMD index with one of three levels of AMD (low, moderate, high), displaying the image of a traffic light (Appendix A, Table A3) [19].

AMD Report

Finally, also as an automatic function, together with the AMD assessment (index and level of AMD), e-12HR provides a report on AMD that includes two numbered lists: (1) Food groups in which the user has successfully fulfilled the MD recommendations (‘compliant’ food groups), and which the user needs to maintain their consumption (highlighted in green); (2) Food groups in which the user has not fulfilled the MD recommendations (‘non-compliant’ food groups), and which require a modification in consumption in order to improve AMD (highlighted in red) [30] (Table 2).

**Figure 3:**
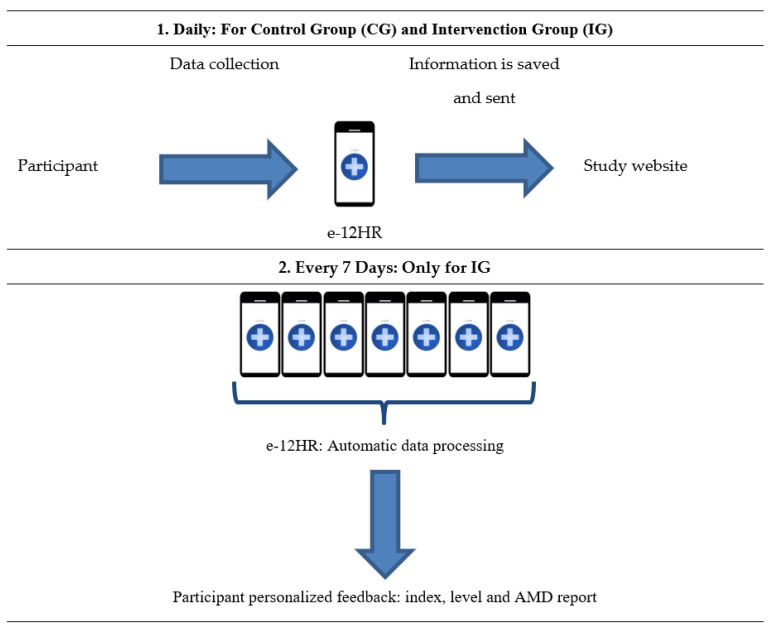
Process Followed Using the e-12HR App during the 28-day Study Period.

Altogether, the process followed in the study was as follows: (1) Every day, the participants recorded their consumption data for a series of food groups through the e-12HR app and, automatically, the app saved data in the participant’s mobile telephone and sent that data to the study website; (2) Every seven days, automatically, the app processed the collected daily information and produced personalized feedback for the user: an AMD assessment (index and level of AMD) and an AMD report (point 2 is only applicable to the IG) (Figure 3). 

The feedback process was carried out at seven days (baseline, week 1) and repeated at fourteen (week 2), twenty-one (week 3), and twenty-eight (week 4) days of monitoring. The AMD report included the two numbered lists of food groups highlighted in green or red so that the user could see, in a simple way, their evolution throughout the study period.

The feedback provided by e-12HR (AMD assessment and AMD report), was available for consultation by participants from the moment it appeared through the “Reports” tab in the app. Furthermore, in the app’s “History” tab, participants could consult the data collected during each day they used e-12HR. 

### 2.5. Follow-Up and Outcome Measures

In order to assess the effect of the intervention (‘feedback’ e-12HR), follow-up was carried out at seven (baseline), fourteen, twenty-one, and twenty-eight days of monitoring.

The main result variable was the change in total score of the AMD index at fourteen, twenty-one, and twenty-eight days of monitoring; while the secondary result variables were the total score of the ADM index, the personal information variables, and the answers to usability rating questionnaire for e-12HR (see Section 2.7. Usability Rating Questionnaire for e-12HR section).

#### Adherence to the Mediterranean Diet

Every seven days during the 28-day study period (at seven (baseline), fourteen, twenty-one, and twenty-eight days of monitoring), the score of AMD index (specifically MDSS index [29]) was calculated manually (for CG and IG) by the research team using the daily data sent by the application to the study website. The research team used the rules shown in Section e-12HR App, (2) Feedback From e-12HR subsection: AMD Assessment

### 2.6. Sample Size Calculation

The sample size was estimated a priori, considering the main result variable. Assuming a standard deviation of 2.9 points in AMD index and a dropout rate of 17.8% throughout the duration of the study—28 days—(from a previous study using e-12HR [25]), a power of 0.8 and an alpha risk of 0.05 in a bilateral test, 161 participants were necessary in each group in order to detect an increase of 1 point in ADM index in the IG compared to the CG. 

The nQuery Advisor Release version 7.0 (Statistical Solutions, Cork, Ireland) was used to calculate the sample size.

### 2.7. Usability Rating Questionnaire for e-12HR

After the 28-day study period, the research team members sent separate e-mails to participants that contained a usability rating questionnaire [24,31,32,33] for the e-12HR app, with five questions about completing the daily e-12HR task (Appendix A, Table A4).

Each participant had to complete this usability rating questionnaire and return it to the same e-mail address.

### 2.8. Ethical Considerations

The study followed the guidelines of the Declaration of Helsinki and was approved by the Research Ethics Committee of the University of Seville, internal code 2813-N-21 (on 30 March 2022).

Written informed consent documents were signed by all patients prior to participating in the study. 

The trial has been registered at ClinicalTrials.gov (identifier NCT05532137).

### 2.9. Statistical Analysis

Discrete variables are presented as a number followed by percentages. Continuous variables are shown using standard deviations and means. 

The data were tested for normality using the nonparametric Kolmogorov–Smirnov test.

For unpaired samples, quantitative variables, Student’s *t*-test or the nonparametric Mann–Whitney U-test was used for unpaired samples, and the chi-square test was used for the comparison of proportions.

For paired samples, two groups: Student’s *t*-test or the nonparametric Wilcoxon test was used for the analysis of quantitative variables (penalizing *p*-values by Bonferroni adjustment for multiple comparisons); three or more groups: ANOVA test or the nonparametric Friedman test was used for the analysis of quantitative variables. 

The results were considered significant if *p*-value < 0.05.

The SPSS statistical software package version 26.0 (SPSS Inc., Chicago, IL, USA) was used for statistical analyses.

## 3. Results

### 3.1. Sample and Adherence to the Study

A total of three hundred and sixty students (from the eight classrooms randomly selected for the study) fulfilled the inclusion requirement (two students were excluded for being diabetic) and signed the informed consent forms. Of them, 74 (35 in CG and 39 in IG) were considered non-responsive since they had completed the task on the app for fewer than 28 days (Figure 1); the data for these participants were not included in the later statistical analysis. The study response rate was 79.4% (286/360): 78.5% (128/163) in CG, and 80.2% (158/197) in IG. 

### 3.2. Personal Information of the Participants

Table 3 shows the personal information of the participants (CG and IG). 

There were no statistically significant differences in the variables studied between the CG and the IG, except in “school”, although in both groups (CG and IG), medical students are the majority (more than 50% of the sample).

There were no statistically significant differences in the study variables between participants who completed the study and those who did not. Those participants who completed the study registered their daily consumption for the included 19 food groups for 8008 days altogether (representing a collected total of 152,152 data points on daily consumption for the food groups).

### 3.3. Effect of the Intervention

For CG and IG, scoring for the AMD index was calculated manually by the research team for each of the weeks during the study monitoring period (using the daily data sent by the app to the study website and the rules shown in Section e-12HR App, (2) Feedback From e-12HR subsection: AMD Assessment.

As for the calculations performed by the research team, the obvious errors resulting during data entry were modified (as the team considered that the data must have been introduced as grams or milliliters instead of standard servings). As an example, on one occasion, a value of 50 was introduced for the question, “How many servings of legumes (lentils, beans, chickpeas, peas, etc.) have you consumed today”? It was considered that this value must indicate consumption of 50 g, which is equivalent to one serving. In any case, the data were modified by the team on only 572 occasions out of 152,152 total registered data points (0.38%).

Table 4 and Table 5 show the AMD indices for CG and IG.

Intragroup modifications: In the CG, the mean score of the AMD index showed few variations throughout the 4 weeks of follow-up (ranging from 7.91, week 1, to 8.20, week 4) (Table 4). There were no significant statistical differences either throughout the 4 weeks of study (Table 4) or in weeks two, three, and four compared to week one (Table 5). In the IG, the mean score of the AMD index ranged from 8.08 (week 1) to 9.63 (week 4) throughout the 4 weeks of follow-up. These differences were statistically significant (Table 4), specifically when comparing weeks two, three, and four with week one (0.54, 1.43, and 1.55 points increase, respectively) (Table 5).

Intergroup modifications: There were significant statistical differences between the AMD indices in the comparison of CG versus IG in weeks two, three, and four, in favor of the IG (0.71, 1.56, and 1.43 points of improvement, respectively) (Table 4).

**Figure 4:**
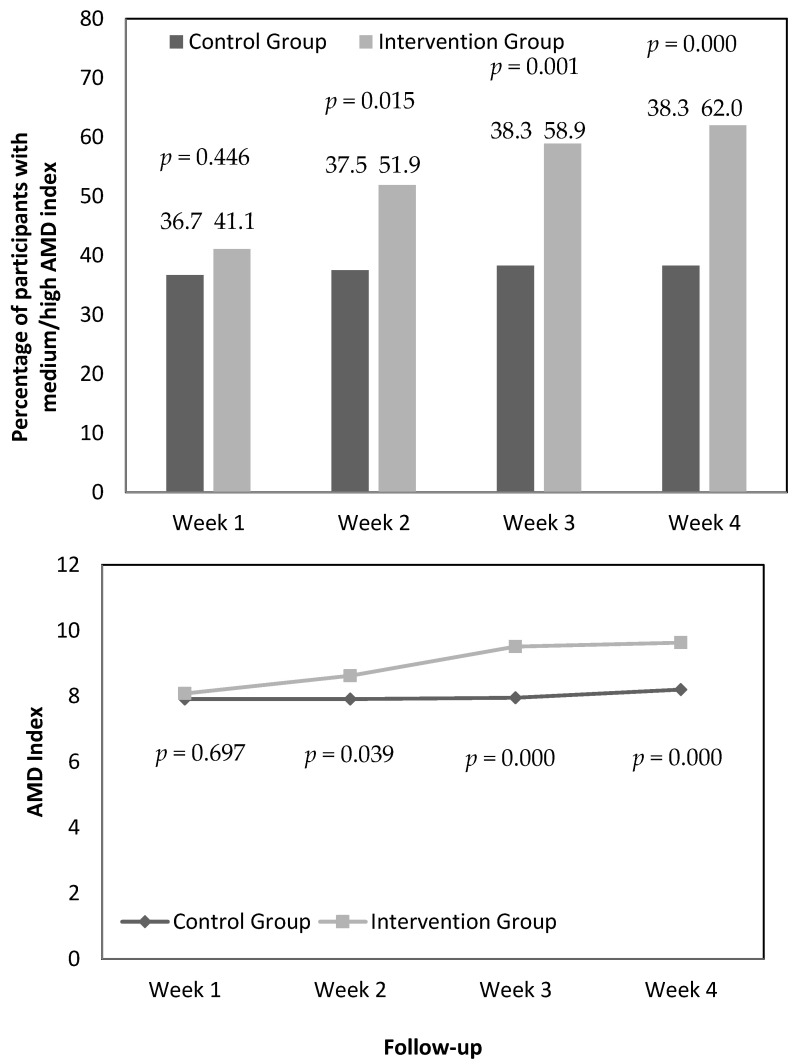
Percentage of Participants with Medium/High Adherence to the Mediterranean Diet (AMD) Index and AMD Indices. *p*-value: intergroup differences (control group versus intervention group) in each of the 4 study weeks, evaluated by the chi-square test (percentage) and evaluated by the Mann–Whitney U-test (AMD index). *p*-value < 0.05 considered significant.

In addition, Figure 4 shows the percentage of participants with medium/high AMD index (MDSS ≥ 9) throughout the 4 weeks of the study in the CG and IG, as well as the AMD index (these last data have already been previously collected in Table 4; however, they are included again in figure format for easy viewing/comparison by readers).

Intragroup modifications: In the CG, the percentage of participants with medium/high AMD index ranged from 36.7% in week one to 38.3% in week four. In the IG, the percentages showed higher differences throughout the 4 weeks of follow-up (from 41.1%, week 1, to 62.0%, week 4) (Figure 4).

Intergroup modifications: At the follow-up in weeks two, three, and four, the percentage of participants with medium/high AMD in the IG increased significantly compared to the CG (an increase of 14.4%, 20.6%, and 23.7%, respectively), similar to the evolution of the mean score of the AMD index (Figure 4).

### 3.4. Usability Rating Questionnaire for e-12HR

The usability questionnaire for e-12HR was completed by 142 participants (62 from the CG and 80 from the IG).

The user responses are shown in Table 6.

Most study participants reported that the e-12HR app was easy to complete (58/62, 93.5%, in the CG and 78/80, 97.5%, in the IG), interesting to complete (53/62, 85.4%, in the CG and 72/80, 90.0%, in the IG) and contained understandable questions/feedbacks (60/62, 96.7%, in the CG and 72/80, 90.0%, in the IG); that they would be willing to complete e-12HR app again (48/62, 77.4%, in the CG and 66/80, 82.5%, in the IG); and that the task took 2–3 min per day to complete (44/62, 71.0%, in the CG and 58/80, 72.5%, in the IG) (Table 6).

## 4. Discussion

The main findings of the study were: One: Intragroup modifications: In the CG, the mean score of the AMD index ranged from 7.91 to 8.20 throughout the 4 weeks of follow-up (no significant statistical differences), and the percentage of participants with medium/high AMD index showed few variations (ranging from 36.7% to 38.3%). In the IG, the mean score of the AMD index ranged from 8.08 to 9.63 throughout the 4 weeks of follow-up (significant statistical differences), and the percentage of participants with medium/high AMD index showed high variations (ranging from 41.1% to 62.0%) (Table 4 and Figure 4). Two: Intergroup modifications: There were significant statistical differences between the two groups in favor of the IG in weeks two, three, and four, in the AMD index (0.71, 1.56, and 1.43 points of improvement, respectively) and in the percentage of participants with medium/high AMD index (increase in the percentage of 14.4%, 20.6%, and 23.7%, respectively) (Figure 4). Additionally, the responses to the usability rating questionnaire for e-12HR (with respect to “easy to complete”, “interesting to complete”, “understandable questions/feedbacks”, “to complete again”, and “time to complete”) were very positive. The usability of the application is a fundamental aspect. In fact, the three principle criteria demanded by healthcare professionals when selecting a “Nutrition & Diet” application for their clients/patients were [3]: ease of use (the ease of use was reflected in the very positive responses to the usability rating questionnaire for e-12HR), free of charge (download is free) and validation (based on the previous version of e-12HR which was previously validated [20,21,22,23,24]).

University students constitute a particularly interesting group to evaluate nutritional interventions to improve the diet for several reasons: (1) The Mediterranean diet is being abandoned, especially among the new generations [15]. (2) Among the younger generations, university students constitute a prominent subgroup since in the 2019/2020 academic year, there were 1309,762 undergraduate students enrolled in Spanish universities [34]. (3) University students are a population at high risk for having unhealthy habits [35] (with low AMD index [25]) as they choose their own food, are very receptive to trends in diet such as slimming diets, skipped meals or consuming snacks, soft drinks, and other new products [36]. (4) University students lead an unhealthy lifestyle and are at risk of preserving these habits throughout adulthood, which represents a great inconvenience for these individuals, especially for students of the Health Sciences [37]. Promoting healthy habits in the population at large, such as maintaining a good diet, is a primary task of these future professionals. Due to this, they should not just know the basics of these habits but practice them as well [38]. Ultimately, universities might provide the ideal forum to reach out to many young adults with nutrition education programs that can positively influence students’ unhealthy eating habits [39]. However, to the best knowledge of the research team, this study has been the first to evaluate an application for mobile telephones to increase AMD among university students (specifically, Spanish university students at the Faculties of Medicine and Pharmacy).

A study that used an application with certain similarities to e-12HR was carried out by Choi et al. [40]. In the study, the participants (patients presenting to the cardiology clinic of an academic medical center) used the application for 6 months, during which the MDSS index was obtained initially, at one month, at 3 months, and at 6 months. The MDSS increased significantly over time for both CG (from 7.1 to 8.4 points) and IG (from 7.2 to 8.8 points), although there was no statistically significant difference in these trends between the two groups. The proportion of participants that achieved high compliance (MDSS ≥ 9) with the MD increased over time from the initial visit to the 6-month visit for both CG (from 18% to 57%) and IG (from 28% to 65%). However, when comparing CG with IG at each time point, there were no significant differences in MDSS ≥ 9 at any of the time points (initial, 1 month, 3 months, and 6 months).

Alonso-Dominguez et al. [41] also used another application with certain similarities to e-12HR. In the study, the participants (patients with type 2 diabetes) used the application for 12 months, during which the MEDAS index (score range 0 to 14 points) was obtained initially, at 3 months, and at 12 months. In the CG, the scores of the AMD index showed no significant variations throughout the study (from 6.9 to 7.0 points). However, in the IG, a significant increase in the ADM index was observed in the follow-up visits with respect to the baseline visit (from 7.2 to 8.5 points). The percentage of participants with suitable ADM index (MEDAS score ≥ 9 points) showed few variations in the CG (ranging from 20% to 21%) and high variations in the IG (ranging from 22% to 50%). Regarding the intergroup modifications which occurred in follow-up visits, at the 3-month follow-up, compared to the baseline follow-up, there was a significant improvement in the AMD index of 2.2 points in favor of the IG. At the same time, this improvement was maintained in the 12-month follow-up visit, with an increase of 1.3 points. At the 3- and 12-month follow-up visits, the percentage of participants with suitable AMD index in the IG increased significantly compared to the CG (an increase of 52% and 29%, respectively).

Another study carried out by Gonzalez-Ramirez et al. [42], who also used a smartphone app with certain similarities to e-12HR, showed that the ADM score was significantly enhanced at month 3 with 1.4 points of improvement (intervention group only—no control group-).

The positive results of these studies [40,41,42] suggest that smartphone applications might represent an opportunity to promote ADM. McAleese et al. [43] carried out another study with the aim of evaluating the quality of commercially available applications (Google Play and Apple App Store) for the MD and the presence of behavioral change techniques (BCTs) used by these tools. The findings of this study suggest that currently available applications might provide information on the MD, but the incorporation of more BCTs is warranted to maximize the potential for behavioral change toward the MD.

The results of the present study, in line with the results obtained by Alonso-Dominguez et al., have shown a significant improvement in both the ADM index and the proportion of participants that achieved high compliance (MDSS ≥ 9) among the participants (university students) who used ‘feedback’ e-12HR (IG) compared to the participants who used ‘non-feedback’ e-12HR (CG). Furthermore, this improvement was observed over shorter follow-up periods than in other studies [40,41,42].

This study has several limitations that should be noted when interpreting the results. First, the exposure to the intervention was short (28 days), and the long-term evolution of the AMD index (once the follow-up period has ended) is unknown. In addition, the main findings of the study were based on patients’ responses: e-12HR application and usability rating questionnaire for e-12HR; e-12HR is a self-reporting method and, as a result, it has limitations inherent in this type of tool (amply described in the bibliography [30,31,44,45,46,47,48]). One of these limitations is its dependence on each participant’s memory and, above all, the difficulty of estimating the size of servings consumed. As far as memory is concerned, e-12HR attempts to reduce this disadvantage by only requiring short-term memory as the task is completed at the end of each day. In fact, the name e-12HR (electronic 12-Hour Dietary Recall) refers to how it differs from a 24-Hour Recall (24 h). While a 24HR registers food group consumption from the day before, e-12HR allows users to record consumption on the same day (normally with 12 h max between food consumption and registration in the app). As for the difficulty of estimating the size of the servings consumed, we should take into account that to assess AMD, it is not required to have a precise estimate of the size of the foods consumed, but rather a precise recognition of the food groups consumed together with an approximate serving estimation [19]; to this end, e-12HR let participants collect data on consumption for all the food groups needed to calculate AMD. Alternative novel methods to determine dietary intake include audio signal processing, image processing, inertial sensing, non-intrusive near-infrared scanning, and also gesture recognition interfacing [49,50,51,52]. Some authors maintain that more research is required to develop these tools, and others, which are more objective and precise, and that resources should be invested to do so [48]. Until these alternatives are available, despite their limitations, digital technologies used for self-report methods can, and must, be developed and used [20] as an improvement over traditional self-reporting methods. This progress constitutes one of the more important challenges that the field of nutritional epidemiology faces at present [32,33,34,35,36,37,38,39,40,41,44,45,46,47,48,49,50,51,52,53]. Finally, another limitation of this study is that women were the majority of the participants (which is actually a reflection of the proportion of male and female students enrolled in the University of Seville’s Schools of Pharmacy and Medicine).

### Future Research Related to the Current Study

Future research will be geared towards evaluating the effectiveness of using ‘feedback’ e-12HR (IG) versus the use of ‘non-feedback’ e-12HR (CG) in increasing AMD index in different situations (e.g., among non-Health Science students, in non-school periods such as summer holidays, and considering different follow-up periods such as more than 28 days), as well as, over the long-term (after a period of time without using e-12HR).

## 5. Conclusions

The results of this study have been promising and suggest that the proposed intervention involving an application for mobile telephones (e-12HR) was relatively effective in the short term at improving the AMD index among university students (a particularly interesting group with which to evaluate nutritional interventions to improve diet but, at the same time, a group not particularly concerned about dietary habits or their associated NCDs). Additionally, the usability of the application could be considered satisfactory.

## Figures and Tables

**Table 1:** e-12HR Data Processing (Every 7 Days).

Scoring of Food Group Calculated on a Daily Basis		
Food Group	Servings per Day	Score (e-12HR)
Fruits	3–6 servings	3
Vegetables	≥6 servings	3
Cereals	3–6 servings	3
Olive oil	3–4 servings	3
Milk and dairy products	2–3 servings	2
Nuts	1–2 servings	2
Fermented beverages	1–2 servings	1
**Scoring of Food Group Calculated on a Weekly Basis**		
**Food Group**	**Servings per Week**	**Score (e-12HR)**
Potatoes	≤3 servings	1
Legumes	≥2 servings	1
Eggs	2–4 servings	1
Fish	≥2 servings	1
White meat	2–3 servings	1
Red/processed meat	<2 servings	1
Sweets	≤2 servings	1
Total maximum score		24

**Table 2:** Example of the Report for Adherence to the Mediterranean Diet (AMD).

Your Global Index of Adherence to the Mediterranean Diet is the Result of Fulfilling the Following Recommendations: 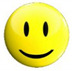	However, to Improve your Global Index of Adherence to the Mediterranean Diet, you Should Make a Little Effort to Follow These Recommendations: 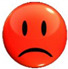
Consume 3–6 servings of cereals (breakfast cereals/pasta/rice/bread) a day (preferably whole grain breakfast cereals/pasta/rice/bread).	Consume 3–6 servings of fruits a day (be sure to wash and peel them first; freshly prepared juices are only equal to one serving of fruit).
2. Do not consume alcoholic beverages. If alcoholic beverages are consumed, it should be performed responsibly and in moderation without exceeding 2 servings a day for men and 1 serving a day for women.	2. Consume ≥6 servings of vegetables a day.
3. Consume ≤3 servings of potatoes a week (preferably cooked or roasted potatoes).	3. Consume 3–4 servings of olive oil a day (preferably extra virgin olive oil).
4. Consume ≥2 servings of fish (and/or shellfish) a week.	4. Consume 2–3 servings of milk and dairy products a day (preferably low-fat preparations and without added sugars).
5. Consume 2–3 servings of white meat a week (be sure to remove the skin and visible fat).	5. Consume 1–2 servings of nuts and/or olives a week (preferably unprocessed, without salt or added sugars).
6. Consume ≤2 times a week Sweets: sugar, candies, pastries, sweetened fruit juices and soft drinks (their consumption should be performed occasionally and moderately—and preferably homemade pastries-).	6. Consume ≥2 servings of legumes a week (replacing a serving of red meat).
	7. Consume 2–4 eggs a week (preferably organic or free-range varieties).
	8. Consume <2 servings of red/processed meat (be sure to eliminate any visible fat).

**Table 3:** Characteristics of the Participants in the Study.

Characteristics	Control Group (CG)		Intervention Group (IG)		
	*n* (%)	Mean (SD)	*n* (%)	Mean (SD)	*p*-Value
Participants who completed the study	128 (100)	- *	158 (100)	-	-
Age (years)	-	20.6 (2.1)	-	20.7 (3.6)	0.453 ^a^
Gender					
Females	91 (71.1)	-	121 (76.6)	-	0.292 ^b^
Males	37 (28.9)	-	37 (23.4)	-	
Schools					
Pharmacy	59 (46.1)	-	40 (25.3)	-	0.000 ^b^
Medicine	69 (53.9)	-	118 (74.7)	-	
BMI (kg/m^2^)	-	22.1 (3.4)	-	21.8 (3.2)	0.432 ^a^
Smoking status					
No	115 (89.9)	-	147 (93.0)	-	0.333 ^b^
Yes	13 (10.2)	-	11 (7.0)	-	
Physical activity status (minutes/week)					
≥150	99 (77.3)	-	117 (74.1)	-	0.520 ^b^
<150	29 (22.7)	-	41 (25.9)	-	

* Not applicable. SD: standard deviation. BMI: body mass index. *p*-value: differences CG versus IG, ^a^ evaluated by the Mann–Whitney U-test and ^b^ evaluated by the chi-square test. *p*-value < 0.05 considered significant.

**Table 4:** Adherence to the Mediterranean Diet (AMD) indices: comparison intra- and intergroups (control group and intervention group).

	AMD Index		
	Control Group (CG)	Intervention Group (IG)	
Number of Week	Mean (SD)	Mean (SD)	*p*
Week 1	7.91 (2.57)	8.08 (2.70)	0.697 ^b^
Week 2	7.91 (2.55)	8.62 (2.73)	0.039 ^b^
Week 3	7.95 (2.49)	9.51 (3.03)	0.000 ^b^
Week 4	8.20 (2.35)	9.63 (3.09)	0.000 ^b^
*p*	0.446 ^a^	0.000 ^a^	

SD: standard deviation. *p*-value in columns: intragroup differences (CG and IG) throughout the 4 weeks of follow-up in the study, ^a^ evaluated by the Friedman test. *p*-value in rows: intergroup differences (CG versus IG) in each of the 4 study weeks, ^b^ evaluated by the Mann–Whitney U-test. *p*-value < 0.05 considered significant.

**Table 5:** Adherence to the Mediterranean Diet (AMD) indices: comparison intragroups (contol group and intervention group).

Control Group (CG)			Intervention Group (IG)		
AMD index					
Number of Week		*p*	Number of Week		*p*
Mean (SD)			Mean (SD)		
Week 1 7.91 (2.57)	Week 2 7.91 (2.55)	0.915	Week 1 8.08 (2.70)	Week 2 8.62 (2.73)	0.006
Week 1 7.91 (2.57)	Week 3 7.95 (2.49)	0.902	Week 1 8.08 (2.70)	Week 3 9.51 (3.03)	0.000
Week 1 7.91 (2.57)	Week 4 8.20 (2.35)	0.172	Week 1 8.08 (2.70)	Week 4 9.63 (3.09)	0.000

SD: standard deviation. *p*-value: intragroup differences (CG and IG) week 1 versus weeks 2, 3 and 4, evaluated by the Wilcoxon test. *p*-value < 0.017 (0.005/3) considered significant (penalizing *p*-values by Bonferroni adjustment for multiple comparisons).

**Table 6:** Study Participants Responses to the Usability Rating Questionnaire for e-12HR.

Questions	Easy to Complete		Interesting to Complete		Understandable Questions/Feedbacks		I Would be Willing to Complete Again	
Group	Control Group,	Intervention Group,	Control Group,	Intervention Group,	Control Group,	Intervention Group,	Control Group,	Intervention Group,
	*n* (%)	*n* (%)	*n* (%)	*n* (%)	*n* (%)	*n* (%)	*n* (%)	*n* (%)
**Options**								
Strongly agree	46 (74.2)	59 (73.8)	19 (30.6)	33 (41.3)	42 (67.7)	44 (55.0)	16 (25.8)	29 (36.3)
Agree	12 (19.3)	19 (23.7)	34 (54.8)	39 (48.7)	18 (29.0)	28 (35.0)	32 (51.6)	37 (46.2)
Neither agree nor disagree	2 (3.2)	2 (2.5)	8 (12.9)	6 (7.5)	2 (3.2)	8 (10.0)	11 (17.7)	10 (12.5)
Disagree	2 (3.2)	0 (0.0)	1 (1.6)	2 (2.5)	0 (0.0)	0 (0.0)	3 (4.8)	4 (5.0)
Strongly disagree	0 (0.0)	0 (0.0)	0 (0.0)	0 (0.0)	0 (0.0)	0 (0.0)	0 (0.0)	0 (0.0)
**Questions**	**Time to Complete**							
	**Control Group,**				**Intervention Group,**			
	***n* (%)**				***n* (%)**			
**Options**								
<1 min/day	0 (0.0)				6 (7.5)			
Approximately 1 min/day	8 (12.9)				10 (12.5)			
Approximately 2 min/day	23 (37.1)				36 (45.0)			
Approximately 3 min/day	21 (33.9)				22 (27.5)			
Approximately 4 min/day	10 (16.1)				4 (5.0)			
5 min/day or more	0 (0.0)				2 (2.5)			

## Data Availability

The data presented in this study are available on request from the corresponding author.

## Appendix A

**Table A1:** Questionnaire Used in e-12HR.

Questions about Food Groups:
1. How many servings of fruits (orange, apple, pear, peach, strawberry, watermelon, etc. -including fresh juice-) have you consumed today? 1 serving = 150–200 g. Homemade measures: 1 serving = A medium-sized piece of apple, pear or orange, a cup of cherries or strawberries, two slices of melon, a glass of natural juice.
2. How many servings of vegetables (tomato, carrot, bell pepper, lettuce, zucchini, etc.) have you consumed today? 1 serving = 150–250 g. Homemade measures: 1 serving = One normal single plate of salad, one normal single plate of cooked vegetables, one large tomato, two carrots.
3. How many servings of breakfast cereals have you consumed today? 1 serving = 20–30 g. Homemade measures: 1 serving = One individual normal bowl.
4. How many servings of pasta have you consumed today? 1 serving = 50–70 g. Homemade measures: 1 serving = One normal individual plate.
5. How many servings of rice have you consumed today? 1 serving = 50–70 g. Homemade measures: 1 serving = One normal individual plate.
6. How many servings of bread have you consumed today? 1 serving = 30–60 g. Homemade measures: 1 serving = Three or four slices of bread or a muffin.
7. How many servings of olive oil have you consumed today (used for salad, to add to bread, or for cooking)? 1 serving = 15 mL. Homemade measures: 1 serving = One tablespoon.
8. How many servings of milk and dairy products (yogurt, fresh cheese, aged cheese) have you consumed today? 1 serving of milk = 200–250 mL. 1 serving of yogurt = 125 g. 1 serving of fresh cheese = 60–80 g. 1 serving of aged cheese = 30–40 g. Homemade measures: 1 serving = A glass of milk, a yogurt, a tub or individual portion of fresh cheese, two or three slices of aged cheese.
9. How many servings of nuts (almonds, walnuts, hazelnuts, etc.) and/or olives have you consumed today? 1 serving = 20–30 g. Homemade measures: 1 serving = A handful of olives (8–10 units), a handful of hazelnuts (18–20 units), three or four walnuts.
10. How many servings of beer have you consumed today? 1 serving = 200 mL. Homemade measures: 1 serving = A beer or a bottle (200 mL). 1.5 servings = A bottle (large bottle), a can or a large beer glass (330 mL).
11. How many servings of wine have you consumed today? 1 serving = 100 mL. Homemade measures: 1 serving = A glass of wine (100 mL).
12. How many servings of potatoes have you consumed today (cooked, roasted, or fried)? 1 serving = 100–150 g. Homemade measures: 1 serving = One large potato or two small potatoes.
13. How many servings of legumes (lentils, beans, chickpeas, peas, etc.) have you consumed today? 1 serving = 50–70 g. Homemade measures: 1 serving = One normal individual plate.
14. How many servings of eggs have you consumed today? 1 serving = One medium egg (50–70 g).
15. How many servings of fish (and/or shellfish) have you consumed today? 1 serving = 100–150 g. Homemade measures: 1 serving = One single regular steak. 0.5 servings = One can of tuna, etc.
16. How many servings of white meat (poultry) have you consumed today? 1 serving = 100–125 g. Homemade measures: 1 serving = One regular single filet, or a chicken leg quarter. 0.5 servings = Two or three slices of chicken or turkey.
17. How many servings of red meat (beef, pork, or lamb) have you consumed today? 1 serving = 100–125 g. Homemade measures: 1 serving = One single regular steak.
18. How many servings of processed meats (hamburgers, sausages) and/or cold cuts (salami, chorizo, cooked ham) have you consumed today? 1 serving = 90–100 g. Homemade measures: 1 serving = a hamburger, a large sausage or two small sausages, eight or ten thin slices of cold cuts (salami, chorizo), four or five thin slices of cooked ham.
19. How many sweets (sugar, candies, pastries, sweetened fruit juices and soft drinks) have you consumed today? Consider the number of units consumed regardless of the serving size.
**Additional questions about physical activities**
20. For how many minutes have you practiced moderate physical activity today? Consider only those moderate physical activities that you practiced for at least 10 min at a time. According to the WHO, these activities require moderate effort, which perceptibly accelerates the heart rate. Examples: fast walking, cycling, dancing, housework, active participation in games and sports with children, etc.
21. For how many minutes have you practiced intense physical activity today? Consider only those intense physical activities that you practiced for at least 10 min at a time. According to the WHO, these activities require a great deal of effort and cause rapid breathing and a substantial increase in heart rate. Examples: running, cycling fast, competitive sports and games, etc.

**Table A2:** Mediterranean Diet Serving Scores (MDSS).

Food Group	Recommendation	Score
Fruit	1–2 servings/main meal	3
Vegetables	≥2 servings/main meal	3
Cereals	1–2 servings/main meal	3
Olive Oil	1 serving/main meal	3
Dairy products	2 servings/day	2
Nuts	1–2 servings/day	2
Fermented beverages	1–2 glass/day	1
Potatoes	≤3 servings/week	1
Legumes	≥2 servings/week	1
Eggs	2–4 servings/week	1
Fish	≥2 servings/week	1
White meat	2 servings/week	1
Red meat	<2 servings/week	1
Sweets	≤2 servings/week	1
Total maximum score		24

Main meal: beakfast, lunch and dinner. Cereals: breakfast cereals, pasta, rice, and bread. Olive oil: used on salads or bread or for frying. Dairy products: milk, yogurt, and cheese. White meat: poultry. Red meat: pork, beef and lamb. Sweets: sugar, candies, pastries, sweetened fruit juices and soft drinks. Fermented beverages: wine and beer (one glass for females and two glasses for males). A score of zero is given when the number of servings/meal, day, or week is higher or lower than the recommendation.

**Table A3:** Levels/scores of Adherence to the Mediterranean Diet (AMD).

Levels of AMD	Score	Colour
Low	0–8	Red
Medium	9–15	Orange
High	16–24	Green

**Table A4:** Usability Rating Questionnaire for e-12HR.

**1. I found e-12HR easy to complete:**
Strongly agree. Agree. Neither agree nor disagree. Disagree. Strongly disagree.
**2. I found e-12HR interesting to complete:**
Strongly agree. Agree. Neither agree nor disagree. Disagree. Strongly disagree.
**3. I found the questions of e-12HR understandable (for control group):** **3. I found the questions/feedback from e-12HR understandable (for intervention group):**
Strongly agree. Agree. Neither agree nor disagree. Disagree. Strongly disagree.
**4. In the future, I would be willing to complete e-12HR again:**
Strongly agree. Agree. Neither agree nor disagree. Disagree. Strongly disagree.
**5. How much time was needed to complete the daily questionnaire on the app:**
Less than 1 min per day. Approximately 1 min per day. Approximately 2 min per day. Approximately 3 min per day. Approximately 4 min per day. 5 min per day or more.
